# 194. Carbapenem Resistance Gene Detection in Positive Blood Cultures Reveals Emergence of NDM-Harboring Organisms and Associated Morbidity and Mortality

**DOI:** 10.1093/ofid/ofad500.267

**Published:** 2023-11-27

**Authors:** Aya Haghamad, Jamie Lemon, Scott Duong, Stefan Juretschko, Vincent Streva

**Affiliations:** Northwell Health laboratories, Little Neck, New York; Northwell Health Laboratories, Lake Success, New York; Northwell Health Laboratories, Lake Success, New York; Northwell Health laboratories, Little Neck, New York; Northwell Health Laboratories, Lake Success, New York

## Abstract

**Background:**

Blood stream infections caused by organisms harboring carbapenemase genes are a significant cause of morbidity and mortality. The prevalence of carbapenemase producing organisms (CPO) has been increasing for several years, with the majority of isolates originating in healthcare settings and containing one of the five most prevalent carbapenemase genes (KPC, OXA-48-like, NDM, VIM, IMP). Management of patients with CPO is particularly challenging and knowledge of the specific carbapenemase gene harbored by these organisms is informative for proper selection of antimicrobial therapy.

**Methods:**

We conducted a retrospective multicenter study of hospitalized patients with blood cultures positive for CPO from January 2021 to present. We used blood culture PCR results to identify the most common carbapenemase genes associated with blood stream infections in our hospital system. Additionally, we evaluated these patients for clinical outcomes including length of hospital stay and mortality.

**Results:**

A total of 61 CPO positive blood cultures from 10 hospitals were included in our study. There was an increase in CPO from 2021 (13 CPO) to 2022 (38 CPO). This increase is maintained year to date in 2023 (9 CPO in Q1) and was primarily driven by the emergence of NDM harboring CPOs in 2022 (19 cultures) continuing into 2023 (5 cultures in Q1). The predominant CPO isolated was *Klebsiella pneumoniae* (40/61, 66%). We observed an increasing trend across the study years in both length of hospital stay (18 days for 2021, 21 days for 2022, 35 days for 2023) and patient mortality (46% for 2021, 54% for 2022, 60% for 2023), though overall clinical outcomes were poor.

**Conclusion:**

Our results suggest that the landscape of CPOs is evolving in the New York City area. Timely reporting and differentiation of carbapenem resistance genes is important to inform changes in antimicrobial therapy. Despite appropriate clinical intervention, mortality rates appear to be increasing for patients with CPO bloodstream infections, likely due to the emergence of NDM-harboring CPOs in 2022.

**Disclosures:**

**All Authors**: No reported disclosures

